# CRISPR-ON-Mediated KLF4 overexpression inhibits the proliferation, migration and invasion of urothelial bladder cancer *in vitro* and *in vivo*

**DOI:** 10.18632/oncotarget.22158

**Published:** 2017-10-27

**Authors:** Xin Xu, Jiangfeng Li, Yi Zhu, Bo Xie, Xiao Wang, Song Wang, Haiyun Xie, Huaqing Yan, Yufan Ying, Yiwei Lin, Ben Liu, Wei Wang, Xiangyi Zheng

**Affiliations:** ^1^ Department of Urology, The First Affiliated Hospital, School of Medicine, Zhejiang University, Hangzhou 310003, Zhejiang Province, P.R. China; ^2^ Department of Urology, Tongde Hospital of Zhejiang Province, Hangzhou 310012, Zhejiang Province, P.R. China

**Keywords:** CRISPR-ON, KLF4, urothelial bladder cancer, proliferation, EMT

## Abstract

Kruppel like factor 4 (KLF4), a transcription factor associated with carcinogenesis and tumor progression, plays an important role in various malignancies. In the present study, we utilized the CRISPR-ON system to upregulate KLF4 expression level and subsequently investigated the effect and mechanism of KLF4 in the carcinogenesis and progression of urothelial bladder cancer (UBC). Immunohistochemistry (IHC) and quantitative RT-PCR (qRT-PCR) were used to evaluate the expression of KLF4. The CpG methylation status of the promoter region was analyzed using bisulfite-sequencing PCR (BSP). CRISPR-ON system comprised sgRNA and dCas9 protein combined with a transcriptional activation domain. The cell proliferation and cell cycle were assessed by CCK-8 assay, flow cytometry and colony formation assay. The cell motility ability was evaluated using trans-well assay. *In vivo* tumorigenesis assay and lung metastasis model were also performed. The KLF4 expression was significantly downregulated in UBC tissues. The high CpG methylation status in the promoter of KLF4 was confirmed using BSP. KLF4 overexpression was successfully achieved via CRISPR-ON system, which inhibited the proliferation and induced G1-phase arrest in T24 cells through the regulation of AKT/p21 signal. Furthermore, enforced expression of KLF4 also abrogated the migration and invasion of T24 cells by suppressing EMT progression. Finally, *in vivo* models indicated that the upregulation of KLF4 could inhibit tumorigenesis and lung metastasis in nude mice. In conclusion, KLF4 overexpression mediated by CRISPR-ON inhibits tumorigenesis and EMT progression in UBC cells, representing a potential therapeutic target, and CRISPR-ON system could be a therapeutic strategy for UBC in the future.

## INTRODUCTION

Urothelial bladder cancer (UBC) is the ninth most frequently-diagnosed cancer worldwide, with an estimated 430,000 new cases diagnosed in 2012 and a mortality rate ranking 13th in terms of deaths [1, 2]. The occurrence and progression of UBC was regarded as results of genetic-environmental interactions. Among all known risk factors, tobacco smoking was considered the main factor, which should be stressed for primary prevention [3]. Although the mechanisms underlying the UBC have been illustrated from different aspects such as genetics, epigenetics and immunology, the precise mechanisms of carcinogenesis remain elusive.

A family of repetitive DNA sequences among the domains of Archaea and Bacteria were firstly detected and defined as clustered regularly interspaced short palindromic repeats (CRISPR) [4]. Subsequently, as illustrated by Barrangou R et al., the specific effect and mechanism of CRISPR was to provide resistance against exogenous virus together with associated CAS genes [5]. The CRISPR/CAS9 system was gradually engineered and utilized as a gene-editing tool in human cells [6, 7]. Initially, CRISPR/CAS9 gene editing system was used to knock down genes for loss-of-function (LOF). Recently, Jaenisch et al. created a CRISPR-ON system comprising a nuclease-dead Cas9 (dCas9) protein combined with a transcriptional activation domain and single guide RNAs (sgRNAs) with sequences complementary to the gene promoter. They have demonstrated that CRISPR-ON can efficiently upregulate exogenous reporter genes in both human and mouse cells [8]. Moreover, CRISPR-ON has the characteristics of robustness and specificity, and can facilitate genome scale gain-of-function (GOF) screening [8, 9].

Kruppel like factor 4 (KLF4), a transcription factor associated with carcinogenesis and tumor progression, has opposite effects and mechanisms in different malignancies. KLF4 was upregulated in osteosarcoma and primary breast ductal carcinoma [10, 11], but downregulated in lung cancer, gastric cancer, hepatoma, prostate cancer, and renal cell carcinoma suggesting its tumor suppressor role [12–16]. In the present study, we introduced a novel gene activation system (i.e., CRISPR-ON) to activate and overexpress KLF4, and then investigated its effects in bladder carcinogenesis and progression.

## RESULTS

### KLF4 is downregulated in UBC cells

**Figure 1:**
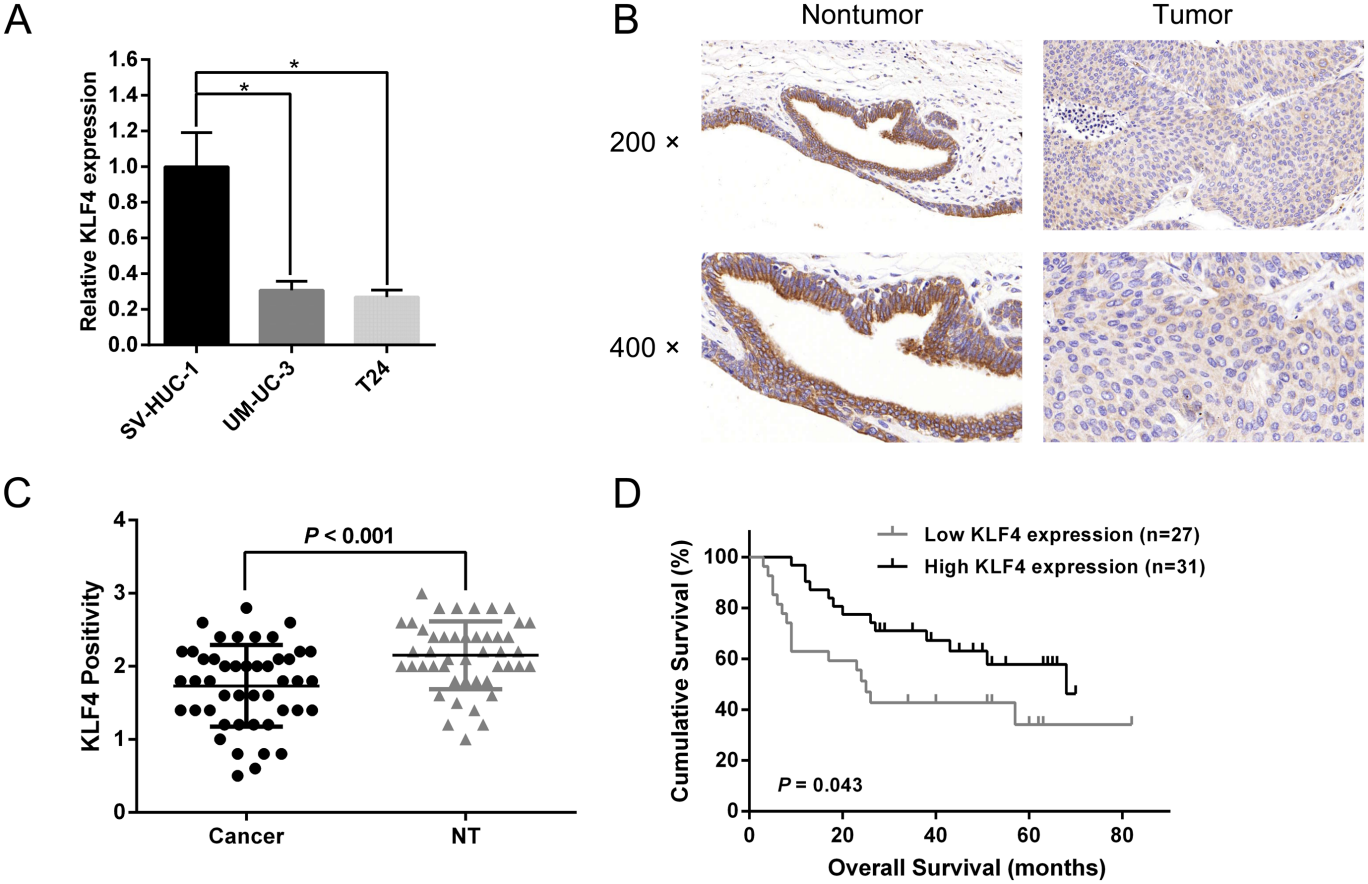
KLF4 is downregulated in UBC tissues and cells **(A)** The results of q-RT-PCR revealed a lower relative KLF4 expression in UBC cells (UM-UC-3 and T24) compared with a normal bladder cell line (SV-HUC-1). **(B)** Typical images of IHC of TMA. KLF4 was localized in the cytoplasm. **(C)** Statistical analysis showed the expression level of KLF4 protein in UBC tissues was aberrantly lower than in adjacent non-tumor tissues. **(D)** Kaplan–Meier survival analysis indicated that the higher protein expression of KLF4 was significantly associated with a higher OS rate in UBC patients. The data were expressed as the means ± S.D. ^*^P < 0.05.

As indicated by the previous published datasets, KLF4 was in a down-regulated expression pattern in UBC compared with normal bladder tissues [17, 18]. To further validate the expression pattern of KLF4 in RCC, we utilized quantitative real-time PCR (qRT-PCR) to quantify the expression levels of KLF4 in a normal bladder cell line (SV-HUC-1) and UBC cell lines (UM-UC-3 and T24). Aberrantly low expression was observed in UM-UC-3 and T24 cells compared with SV-HUC-1 cells (Figure 1A). To further evaluate the expression and subcellular localization of KLF4 in UBC tissue and adjacent non-tumor tissue, we analyzed the immunohistochemistry (IHC) results which showed KLF4 was located in cytoplasm (Figure 1B). Besides, statistical analysis revealed the significantly low expression of KLF4 in UBC tissue compared with non-tumor tissue (*P* < 0.001, Figure 1C). A survival analysis was conducted to evaluate the prognostic value of KLF4. Kaplan-Meier survival curves indicated that the KLF4 expression was closely associated with the overall survival (OS) rate in UBC patients (Figure 1D). All above results suggested that KLF4 might play a crucial role in the progression of UBC.

### CRISPR-ON mediated KLF4 overexpression in T24 cells

**Figure 2:**
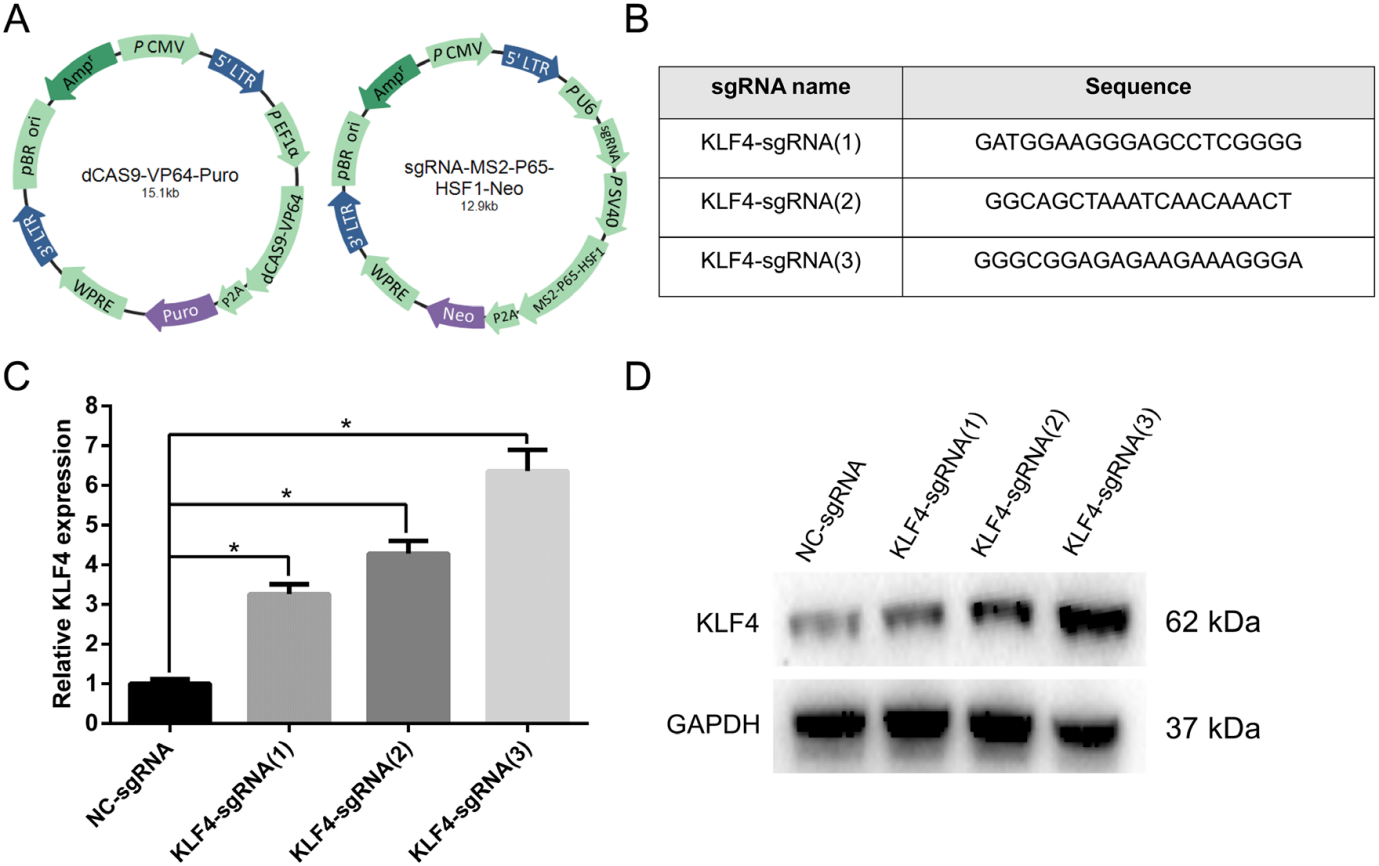
The CRISPR-ON system upregulated the expression level of KLF4 **(A)** The constructed lentiviral plasmid diagrams were shown: lenti-Dcas9-vp64-Puro and lenti-sgRNA-MS2-P65-HSF1-Neo. **(B)** Three sgRNA sequences were designed. **(C)** The results of qRT-PCR suggested that the relative mRNA expression level of KLF4 was obviously upregulated by CRISPR-ON system. **(D)** The Western blot analysis showed the upregulation of KLF4 protein expression by CRISPR-ON system. The data were expressed as the means ± S.D. ^*^ *P* < 0.05.

In the present study, we introduced a novel technique to activate the expression of KLF4 in UBC, namely CRISPR-ON system. CRISPR-ON related plasmids, including Lenti-Dcas9-vp64-Puro and Lenti-sgRNA-MS2-P65-HSF1-Neo, were purchased from Shanghai Genechem Co., Ltd, which assembled the synergistic activation mediator (SAM) complex with a sgRNA targeting the promoter of KLF4 (Figure 2A). We designed three sgRNAs with the following sequences: KLF4-sgRNA(1): GATGGAAGGGAGCCTCGGGG; KLF4-sgRNA(2): GGCAGCTAAATCAACAAACT; and KLF4-sgRNA(3): GGGAGAGAAGAAAGGGA (Figure 2B). The upregulation level of KLF4 mediated by CRISPR-ON was analyzed in both mRNA and protein levels, and the results indicated KLF4 was significantly upregulated in T24 cells (Figure 2C and 2D) and KLF4-sgRNA(3) showed the best activation effect.

### KLF4 overexpression mediated by CRISPR-ON inhibited cell proliferation and induced G1-phase arrest in T24 cells

**Figure 3:**
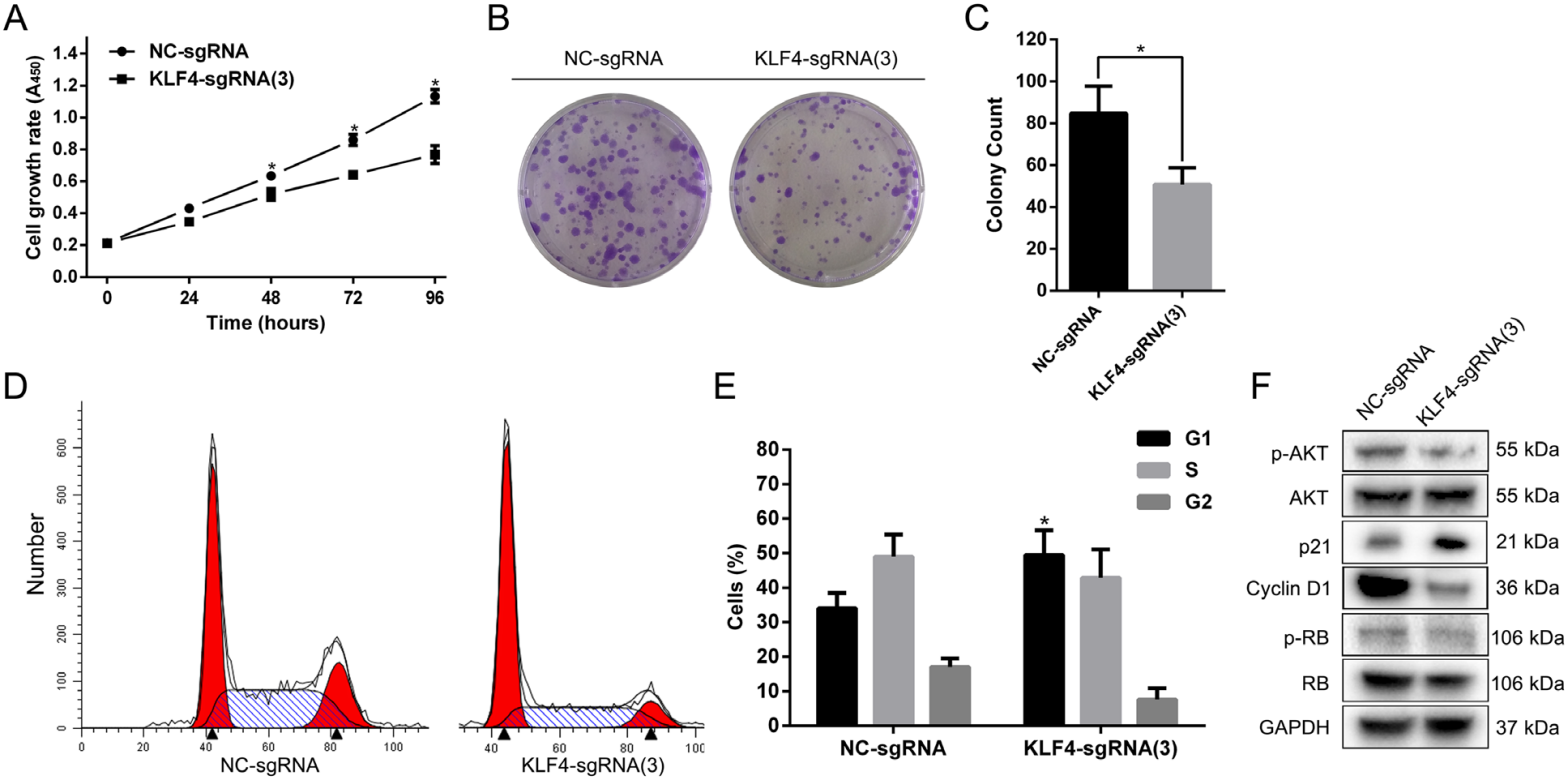
Overexpressed KLF4 abrogated the proliferation and induced G1-phase arrest in T24 cells **(A)** The cell growth curve showed the retard of T24 cells treated with SAM fused with sgRNA-KLF4(3) compared with NC. **(B)** The colony formation assay also indicated the reduced colony rate in T24 cells treated with SAM fused with sgRNA-KLF4(3). **(C)** The colony count was calculated. **(D)** The cell cycle assay revealed G1-phase arrest in T24 cells treated with SAM fused with sgRNA-KLF4(3). **(E)** The percentages of cells in different phases were calculated. **(F)** Overexpressed KLF4 inhibited the proteins associated with the AKT/p21 signal. The data were expressed as the means ± S.D. ^*^ *P* < 0.05.

To analyze the effect of KLF4 on proliferation, we utilized the SAM complex and KLF4-sgRNA(3) to activate the expression of KLF4. The cell growth curve, colony formation assay and cell cycle flow cytometry assay in T24 cells were performed. T24 cells treated with SAM fused with KLF4-sgRNA(3) significantly inhibited cell growth and colony formation rate compared with cells treated with NC-sgRNA (Figure 3A, and 3C). Furthermore, KLF4 overexpression induced G1-phase arrest in T24 cells (Figure 3D and 3E). Western blot revealed p-AKT, CCND1 and p-RB protein level were downregulated, whereas p21 protein level was upregulated (Figure 3F).

### KLF4 overexpression mediated by CRISPR-ON abrogated the invasion and migration of T24 cells

**Figure 4:**
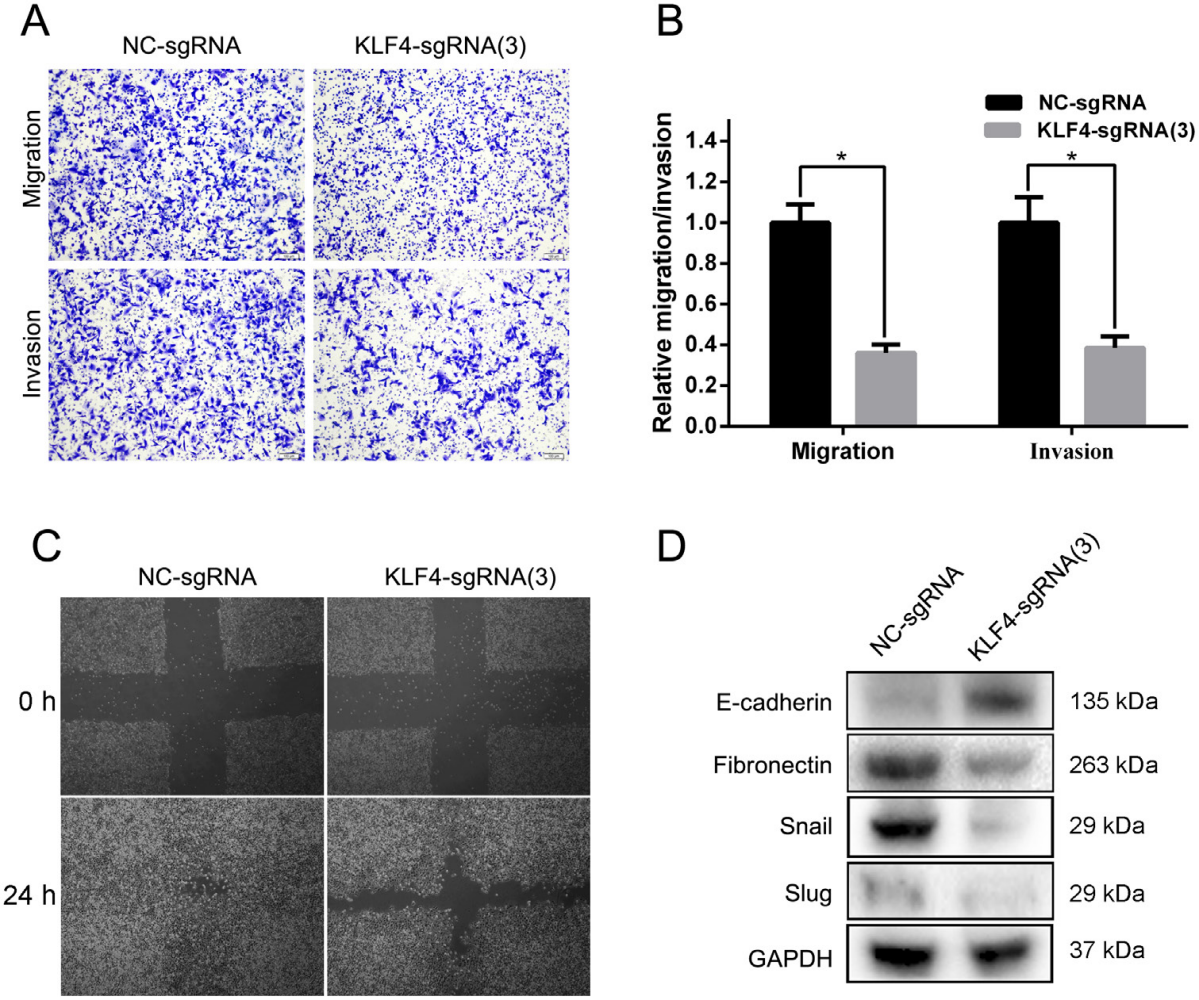
Overexpressed KLF4 inhibited invasion and migration in T24 cells **(A)** The results of trans-well assay (representative micrographs are presented) demonstrated the inhibition of invasion and migration in the sgRNA-KLF4(3) group. **(B)** The relative invasion and migration rate was calculated. **(C)** A wound healing assay indicated that the overexpression of KLF4 in T24 cells resulted in a retardation of wound closure compared with the control group. **(D)** EMT progression was suppressed in the sgRNA-KLF4(3) group. The data were expressed as the means ± S.D. ^*^ *P* < 0.05.

To evaluate the effect of KLF4 on invasion and migration, we also utilized the SAM complex and KLF4-sgRNA(3) to activate the expression of KLF4 in T24 cell lines. The results of trans-well assay suggested a significant inhibition of invasion and migration in cells treated with SAM fused with KLF4-sgRNA(3) compared with cells treated with NC-sgRNA (Figure 4A and 4B). A wound healing assay indicated that the overexpression of KLF4 in T24 cells resulted in a retardation of wound closure compared with the control group (Figure 4C). Western blot analysis showed the downregulation of EMT associated proteins Fibronectin, Snail, and Slug, and the upregulation of E-cadherin (Figure 4D).

### KLF4 overexpression mediated by CRISPR-ON inhibited the growth of tumor xenografts and lung metastasis *in vivo*

**Figure 5:**
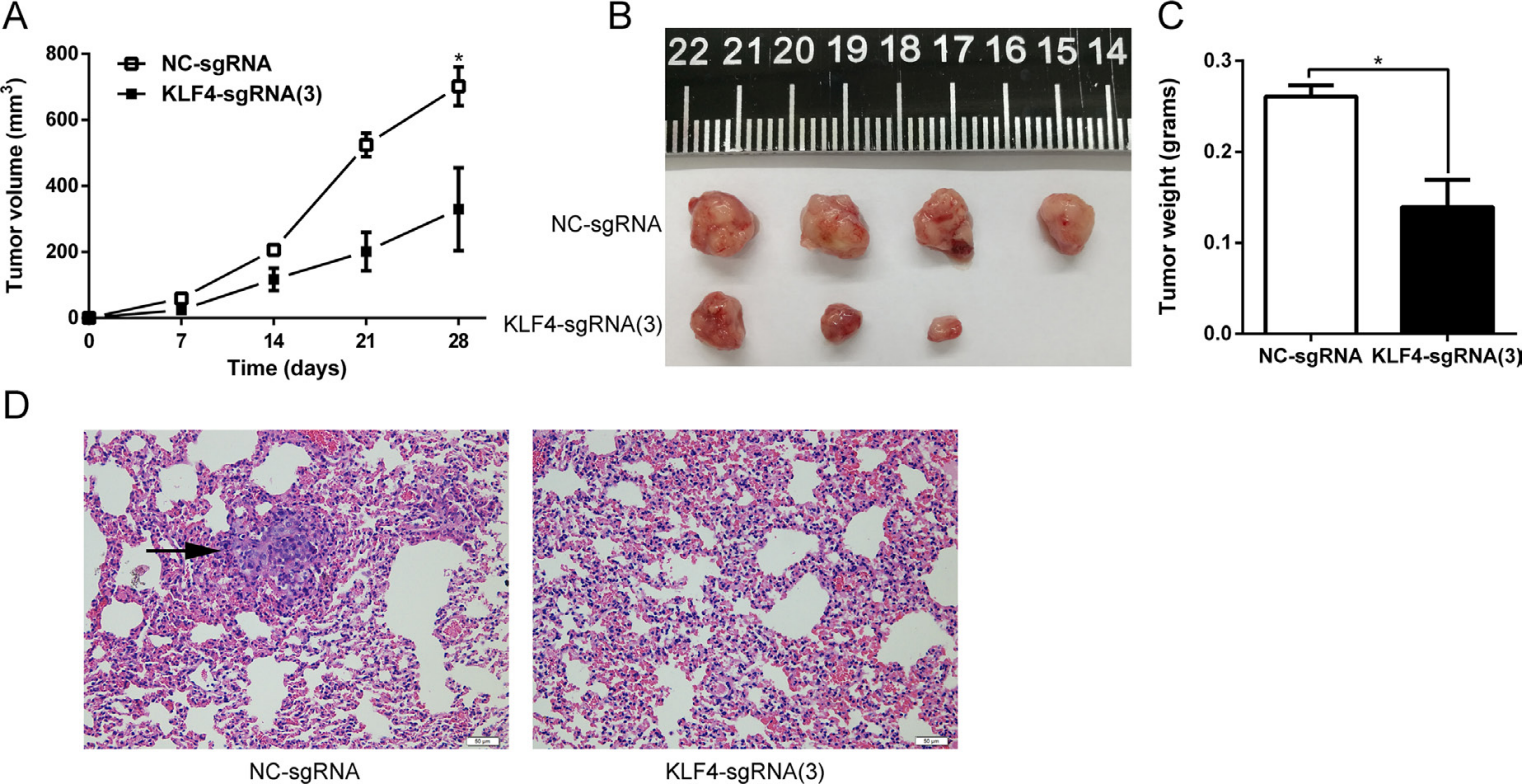
KLF4 inhibited tumor growth and lung metastasis *in vivo* **(A)** The growth of tumor xenografts originated from T24-KLF4(3) cells was retarded compared with that in the NC group. **(B)** The tumor xenograft was dissected from the sacrificed naked mice. **(C)** The tumor weight was measured and compared. **(D)** H&E staining of mouse lungs. Lung metastatic foci was indicated with a black arrow. The data were expressed as the means ± S.D. ^*^ *P* < 0.05.

Tumor xenografts and a lung metastasis model were established to evaluate tumorigenesis *in vivo* following *in vitro* studies confirming the tumor suppressor role of KLF4 in UBC. T24-NC and T24-KLF4(3) cells were separately injected subcutaneously into the flank of nude mice to establish the tumor xenografts. Tumor size measured weekly was significantly retarded in xenografts originating from T24-KLF4(3) cells *(P* < 0.05) (Figure 5A). The mice were sacrificed 4 weeks later, and tumor xenografts were dissected and weighted. The results also indicated a significant tumor weight difference induced by overexpression of KLF4 (*P* < 0.05) (Figure 5B and 5C). Similarly, lung metastasis model was constructed by tail vein injection of T24-NC and T24-KLF4(3) cells. After 6 weeks, the nude mice were sacrificed for histopathology, and the results revealed that the metastasis foci in 4 mice of in NC-group (n=5), whereas no metastasis foci were detected in mice of T24-KLF4(3) group (n=5) (Figure 5D). Taken together, these *in vivo* experiments also indicated that KLF4 overexpression via CRISPR-ON inhibited the growth and metastasis of UBC cells.

### High CpG methylation status in the promoter of KLF4 was confirmed with Bisulfite-sequencing PCR (BSP)

**Figure 6:**
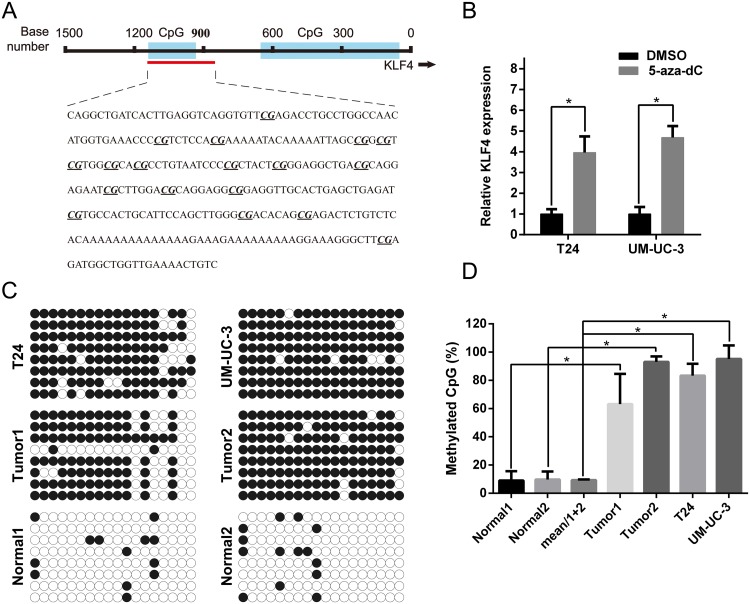
High CpG methylation status in the promoter of KLF4 was confirmed using BSP **(A)** The regions analyzed using BSP were indicated. **(B)** qRT-PCR indicated that 5-aza-dc treatment promoted the expression of KLF4 in both T24 and UM-UC3 cells. **(C)** Tumor tissues and cell lines (T24 and UM-UC-3) possessed higher methylation sites than normal bladder tissues. **(D)** The methylated CpG rate was calculated and compared. The data were expressed as the means ± S.D. ^*^ *P* < 0.05.

Previous studies have indicated that the epigenetic modification might play a great role in KLF4 expression [17, 19]. We uesd CpG Island Searcher program (http://www.urogene.org/methprimer/) to identify the CpG islands in the 1500 bp region upstream of KLF4 (Figure 6A). The expression of KLF4 in T24 and UM-UC-3 cells were significantly upregulated after treatment with 5-aza-CdR, a DNA methyltransferase inhibitor (Figure 6B). The predicted CpG methylation status was identified by BSP, revealing that T24 and UM-UC-3 cells, and tumor tissues, were aberrantly hyper-methylated in CpG islands of the KLF4 promotor region compared with para-tumor normal tissue (Figure 6C). The percentage of methylated CpG islands was calculated and all results were statistically significant (Figure 6D).

## DISCUSSION

UBC is the ninth most frequently diagnosed cancer worldwide [1]. Traditional treatment options for UBC have been merely confined to operation, radiotherapy and chemotherapy, but the clinical efficacy of these strategies was not completely satisfying and quite limited. Recently, a surging number of potential therapeutic strategies have emerged. The targeted drug anti-PDL-1 MPDL3280A obtained rapid and ongoing responses in patients with metastatic UBC [20, 21].

A number of studies have confirmed that gene editing technology showed prominent advantages of laser-like precision [22, 23]. CRISPR/Cas9 technology as a third-generation of gene editing technology features specificity and efficiency, and has become the main tool for genome editing in many laboratories. Owing to the widespread use in comprehensive fields, CRISPR/Cas9 technology promises to be a possible approach for human gene therapy [24]. The engineered CRISPR-ON system, comprising a dCas9 protein combined with a transcriptional activation domain and sgRNAs, functions as a new-rising genome activation strategy, which was confirmed to activate IL1RN, SOX2, OCT4 and INS genes [8, 19], and promises to be a valuable tool for gene precision therapy in the future.

KLF4 is a zinc-finger transcription factor expressed in epithelial cells of organs, such as lung, skin, and gastrointestinal tract cells [25, 26]. KLF4 is critical for the induction of pluripotency and stem cell maintenance [27]. The potential effects of KLF4 in tumor carcinogenesis were variable in different malignancies. Previous study has elucidated that the deficiency of KLF4, as an oncogene, inhibited breast cancer pulmonary metastasis in mice [28]. Further studies identified KLF4 and KLF5 as cooperating protumorigenic factors and critical participants in resistance to lapatinib, providing the theoretical basis for combining anti-MCL1/BCL-XL inhibitors with conventional HER2-targeted therapies [29]. By contrast, Li et al. reported that the inactivation of KLF4 in villin-positive gastric progenitor cells induced the transformation of the gastric mucosa and tumorigenesis in the antrum of mice [30]. KLF4 was also shown to induce cell fate changes and the downregulation of this protein promoted pancreatic carcinogenesis [31]. While investigating the mechanism of KLF4 downregulation, Filarsky et al. observed that KLF4 was down-regulated in chronic lymphocytic leukemia owing to aberrantly hyper-methylated status of promoter, and this effect could be reversed by inhibition of NOTCH signaling [19]. To sum up, considerable evidences have indicated that KLF4 is intimately involved in the carcinogenesis and cancer progression.

The role of KLF4 in UBC has been preliminarily explored and associated with tumor progression and early recurrence in a previous study [17]. In contrast, we also performed a mouse lung metastasis model assay and explored the association of KLF4 with OS rate. Most importantly, in the present study we applied the CRISPR-ON system as an efficient genome activation tool to activate the expression of KLF4 in UBC. To our best knowledge, this is the first study to explore the therapeutic effects of the CRISPR-ON system in human cancers.

Our study confirmed that KLF4 expression was significantly downregulated in human UBC tissues and associated with poor OS. The hyper-methylated status of the KLF4 promoter was identified with BSP, consistent with the results of two previous studies [17, 19]. Furthermore, KLF4 overexpression via CRISPR-ON system inhibited proliferation and induced G1-phase arrest by the regulation of AKT/p21 signal, which was famous p53 targeted genes and maintained survival of UV-induced DNA-damaged cells [32]. Migration, invasion and EMT progression were also suppressed by the upregulation of KLF4. Importantly, KLF4 overexpression mediated by CRISPR-ON system inhibited the growth of tumor xenografts and lung metastasis *in vitro*. Overall, these results supported a tumor suppressor role for KLF4 in UBC.

Prior to the gene activation mediated by CRISPR-ON, Li et al. [33] described small dsRNAs inducing transcriptional activation in human cells, which was defined as RNA activation (RNAa). These authors have identified several dsRNAs that activate gene expression (E-cadherin, p21, VEGF) by targeting noncoding regulatory regions in gene promoters [33]. Subsequently these authors reported that miR-373 induced the expression of genes with complementary promoter sequences [34]. In previous studies, we showed that the upregulation of PAWR mediated with small dsRNAs induced cell apoptosis in prostate cancer [35]. And the overexpression of p16 induced by the enforced expression of miR-877-3p inhibited the proliferation and tumorigenesis of UBC [36]. Although RNAa can mediate gene expression, the application is confined to its’ several defects. DNA hypermethylation in promoter region was reported as an obstacle for RNAa, and CpG islands are generally excluded in the target sequences of small dsRNAs. Moreover, non-specific gene activation appeared sometimes due to its off-target effects [33, 37, 38]. Compared with RNAa, increasing evidence indicates that the CRISPR-ON system has high specificity and low incidence of off-target effects [39–41]. In the present study, KLF4 overexpression mediated by CRISPR-ON was not restricted by the hypermethylated status of the promotor, and the robust activation of KLF4 was acquired.

## MATERIALS AND METHODS

### Cell lines and cell culture

The human bladder cancer cell lines UM-UC-3 and T24 and normal bladder cell SV-HUC-1 were purchased from the Shanghai Institute of Cell Biology (Shanghai, China) and were cultured in RPMI 1640 medium supplemented with 10% heat-inactivated fetal bovine serum under a humidified atmosphere of 5% CO_2_ at 37°C.

### CRISPR-ON system and transfection

SAM complex was obtained from Shanghai Genechem Co., Ltd, containing the lenti-Dcas9-vp64-Puro and lenti-sgRNA-MS2-P65-HSF1-Neo. Trypsin-digested T24 cells (5 × 10^4^) were plated into 6-well, when the confluence of cells was 30%, and the cells were subsequently infected with an appropriate concentrated lenti-dCAS9-VP64-Puro according to MOI followed by incubation for 3 days at the atmosphere of 5% CO_2_ at 37°C. A suitable concentration of puromycin was added for positivity screening for 7 days and then maintained at a low concentration for sustained incubation. Lenti-sgRNA-MS2-P65-HSF1-Neo infection and G418 positivity screening were continued. In final, after lentivirus based plasmid transfection and screening twice, a T24 cell line stably overexpressing KLF4 (transfected with KLF4-sgRNA) was generated, with the negative control (transfected with NC-sgRNA).

### RNA isolation and qRT-PCR

The mRNA was extracted with RNAiso Kit for total RNA (TaKaRa, Japan) and immediately transcribed into cDNA using One Step PrimeScript miRNA cDNA Synthesis Kit (TaKaRa, Japan). Subsequently, the ABI 7500 FAST Real-Time PCR System (Applied Biosystems, USA) and a SYBR Green PCR Kit (TaKaRa, Japan) were applied to detect the Ct value of KLF4. GAPDH was used as an endogenous reference to calculate the relative expression of KLF4 with 2^-ΔΔCt^ (delta-delta-Ct algorithm) method. The following primers used are listed as follows: GAPDH-F: 5’AAGGTGAAGGTCGGAGTCA3’, GAPDH-R: 5’GGAAGATGGTGATGGGATTT3’. KLF4-F: 5‘ACCCACACTTGTGATTACGC3’, KLF4-R: 5‘CCGTGTGTTTACGGTAGTGC3’.

### Western blot analysis

The proteins were extracted from the treated cells using the RIPA lysis buffer method, and relatively quantified using BCA Protein Assay kit. The extracted proteins were loaded onto 10% SDS-polyacrylamide gels and electrophoresed fully. Subsequently, the separated proteins were transferred to a PVDF membrane using a wet transfer method. The membrane was blocked with 5% fat-free milk for 1 hour, and subsequently incubated with primary antibody (at 1:1000 ratio) at 4°C overnight. The membrane was washed 3 times (totally 30 min) with TBS-Tween buffer, and treated with secondary antibody (diluted with diluent as 1:5000 ratio) for 1 hour at room temperature. After washing 3 times with TBS-Tween buffer, the protein level was detected with enhanced chemi-luminescence (ECL) system (Pierce Biotechnology Inc., USA). The primary antibodies used are listed as follows: anti-GAPDH, anti-KLF4, anti-AKT, anti-p-AKT, anti-p21, anti-CCND1, anti-RB, anti-p-RB (Cell Signaling Technology, USA), anti-E-cadherin, anti-Fibronectin, anti-Snail, anti-Slug (Epitomics, USA).

### Cell growth assay

Generally, 4 × 10^3^ T24 NC cells and T24 KLF4 sgRNA(3) cells were plated in each well of a 96-well plate separately, followed by the incubation of 0, 24, 48, 72, 96h at atmosphere of 37 °C and 5% CO_2_. The cell viability and growth rate were detected using a CCK-8 assay kit (Dojindo Laboratories, Japan).

### Cell cycle analysis with flow cytometry

T24 NC and T24 KLF4 sgRNA(3) cells were collected after trypsin digestion and fixed with 75% ethanol overnight at 4 °C. After the fixation, the cells were washed with PBS 2 times and treated with RNase A and propidium iodide (50 μg/ml) for 30 min. Then BD LSRII Flow Cytometer System with FACSDiva software (BD Bioscience, USA) was used to analyze the cell cycle and the data were modified using ModFit LT 3.2 software (Verity Software House, USA).

### Colony formation assay

T24 NC cells and T24 KLF4 sgRNA(3) cells were collected with trypsin digestion and seeded onto 6-well plates, at approximately 500 cells per well, followed by the incubation for 2 weeks. Subsequently, methanol and 0.1% crystal violet were used for fixing and staining, respectively.

### Cell migration and invasion assay

Trans-well chambers and matrix gel were used to evaluate cell motility. The matrix gel was plated on the bottom of the trans-well chamber, and subsequently approximately 3×10^4^ T24 NC and T24 KLF4 sgRNA(3) cells suspended in 0.2 ml serum-free medium were placed onto the surface layer of matrix gel. The entire chamber was placed into a 24-well plate, and 600 μL RPMI-1640 medium with 10% FBS was added to space between the chamber and well. After incubation for 24 hours at 37 °C, we detected the invasion rate using methanol and 0.1% crystal violet treatment. The migration rate operation omitted the matrix gel step.

### IHC analysis

We acquired the tissue microarrays (TMAs) from Xinchao Biotech, Shanghai, China, comprising 46-paired tumor and non-tumor parts and 13 cases without corresponding non-tumor tissues. Antigen retrieval was performed after heating the slides in sodium citrate buffer (10 mM, pH6.0). The slides were incubated with anti-KLF4 (Cell Signaling Technology, USA) overnight at 4 °C after blocking with bovine serum albumin (Sango Biotech, China). Subsequently a secondary antibody was added to slides for incubation for 1 h at room temperature. A DAB solution was used for brown color development. Positivity rate of KLF4 was semi-quantified considering both the intensity and proportion of positive cells.

### DNA methylation analysis and 5-aza-CdR treatment

RNA was isolated using a RNAiso kit and qRT-PCR was conducted to evaluate the expression of KLF4, after T24 and UM-UC-3 cells were treated with 5 μM 5-aza-2′-deoxycytidine (5-aza-CdR, Sigma A3656, USA) for 4 days. BSP was started with bisulfite conversion, and then the CpG islands of KLF4 were amplified using the following primers 5′- TAGGTTGATTATTTGAGGTTAGGTG-3′ (forward) and 5′-AACAATTTTCAACCAACCATCTC-3′ (reverse). The PCR products were cloned into the pUC18 T-vector. Bacterial amplification was performed and eight clones were subjected to DNA sequencing (Sangon, China).

### *In vivo* tumorigenesis assay and lung metastasis model

Male BALB/c-nude mice (4 weeks old) were purchased from the Shanghai Experimental Animal Center, Chinese Academy of Sciences, Shanghai, China. For the *in vivo* tumorigenesis assay, 1×10^6^ T24-NC and T24-KLF4(3) cells were separately injected subcutaneously into the flank of naked mice. Tumor size (V = (width^2^ × length × 0.52)) was measured weekly by vernier caliper. For the construction of the lung metastasis model, 10 naked mice were randomly distributed into two groups. 5×10^5^ T24-NC and T24-KLF4(3) cells were injected into the tail vein of naked mice according to groups. The mice were sacrificed 6 weeks, and their lung lobes were dissected for histopathology. All animal studies and manipulations were performed in accordance with the institutional guidelines approved by First Affiliated Hospital, School of Medicine, Zhejiang University.

### Statistical analysis

The data were expressed as the means ± S.D.. Differences between groups were estimated using Student's t-test. OS rates were calculated according to the Kaplan–Meier method with log-rank test. All analyses were conducted using SPSS 16.0 software (IBM, USA) and significance was defined as a two-tailed value of p<0.05.

## CONCLUSION

This study suggested that KLF4 overexpression mediated by the CRISPR-ON system inhibits proliferation, migration and invasion by regulating AKT/p21 signal and EMT progression, representing a potential therapeutic target, and CRISPR-ON system could be a therapeutic strategy for UBC in the future.
